# Multilingualism educational attainment and cognitive development in UK adolescents

**DOI:** 10.1038/s41539-026-00411-z

**Published:** 2026-04-01

**Authors:** Roisin C. Perry, Roberto Filippi, Mireille B. Toledano, Iroise Dumontheil, Chen Shen, Michael S. C. Thomas

**Affiliations:** 1 University College London, Institute of Education, London, UK; 2 Centre for Educational Neuroscience, London, UK; 3 School of Human and Social Sciences, University of West London, London, UK; 4 School of Psychological Sciences, Birkbeck University of London, London, UK; 5 Multilanguage and Cognition Lab, UCL, Institute of Education, London, UK; 6 Department of Epidemiology and Biostatistics, School of Public Health, Imperial College London, London, UK; 7 Melbourne School of Psychological Sciences, University of Melbourne, Melbourne, Australia

**Keywords:** Education, Psychology, Psychology

## Abstract

Evidence for educational or cognitive advantages in multilinguals is mixed and may reflect socioeconomic or cultural confounds. Using 1673 UK adolescents from the SCAMP cohort, we examined educational attainment at ages 11 and 16 and change in cognitive control between these time points, comparing simultaneous multilinguals, children learning English as a second language, and monolinguals while adjusting for relevant covariates. Simultaneous multilinguals showed a small but reliable attainment advantage over monolinguals at both ages, whereas cognitive differences appeared only in cross-sectional analyses. Children learning English as a second language showed lower attainment at 11, steeper gains, and higher attainment by 16, with no evidence of cognitive differences. Mediation analyses indicated that attainment advantages were largely independent of cognitive measures, suggesting that educational outcomes and cognitive differences should be decoupled. These findings inform policy discussions on multilingual education.

## Introduction

The impact of multilingualism on cognition and on educational outcomes remains a pivotal yet contentious topic within developmental psychology and neuroscience. Early 20th century research suggested that learning multiple languages could impede cognitive development and academic performance [see 1 for a review]. This influenced generations of educational policy and parental choices and fostered a monolingual bias in educational systems worldwide [see 2 for a review]^1,2^.

More recent research indicates that multilingualism may affect cognitive abilities, with suggestions of higher scores in tests of executive functions such as working memory, cognitive flexibility, and attention control [see 3 for a review]. For education, researchers distinguish between children speaking more than one language from birth (henceforth *simultaneous multilinguals*) and those learning a second language (henceforth *multilingual learners*), for example, children from immigrant families learning the language of the country to which they have moved. Recent evidence from the United Kingdom suggested that at the end of primary education, simultaneous multilinguals outperformed monolinguals in national tests, while multilingual learners lagged behind monolinguals; but by age 16, both multilingual groups outperformed monolinguals^3^.

A tempting inference would be that the observed educational advantage of multilingualism is due in part or in full to the cognitive differences. However, to date, this hypothesis has been difficult to evaluate for several reasons. First, the exact nature of the cognitive difference is still debated, with even meta-analyses yielding inconsistent findings^4–6^. Different proposals for the locus of the effect range from greater efficiency of specific cognitive processes involved in controlling two languages (such as inhibiting the context-irrelevant language or switching between languages) which then transfers to non-linguistic tasks, to enhancement of a putative domain-general resource such as selective attention, to more diffuse effects of cognitive stimulation, such as that sometimes linked with variations in socioeconomic status^7^ (see also^8,9^ for discussion).

Second, it is difficult to discern the unique effects of multilingualism on cognitive development and educational outcomes because multilingual status is not conferred at random. There may be other differences between multilinguals and monolinguals that explain cognitive and educational differences between them. Some researchers have argued that socioeconomic status (SES) is such a confound^10,11^. Alternatively, cultural differences, such as in attitudes to education or opportunities for different types of cognitive or social stimulation, may produce the effects. As Bialystok^8^ comments, ‘bilingual experience is complex and there are a range of cognitive, linguistic, and social aspects to that experience that may contribute to the cognitive effects found for bilingualism. No particular aspect of language use has been found to account for these effects’ (p.4).

Ideally, to address the role of cognitive skills in producing the multilingual educational advantages, we need a representative sample of multilinguals and monolinguals large enough that the contribution of language status and potential confounds such as SES and cultural factors can be teased apart. Moreover, use of a longitudinal design would further attenuate the influence of factors incidentally associated with multilingual status as it would focus on changes within individuals. In this study, we present the results of a cohort study of 1673 adolescents in the UK, tracked longitudinally from the end of primary school to secondary school examinations, who undertook cognitive tests between those time points to measure their growth in executive function skills and non-verbal reasoning. This study builds on our earlier SCAMP analysis^3^ by incorporating longitudinal data, a refined multilingual classification, and a broader set of cognitive and educational predictors. Adolescence is an understudied phase with respect to multilingual effects on cognition^12^ but is key for two reasons: advanced skills in cognitive control are still developing^13^ and children are completing national examinations that are important for their future educational and professional prospects. We identified and controlled for the influence of SES and cultural factors (indexed by ethnicity) to address the question of whether multilingualism confers a cognitive difference in the teenage years and whether this difference is responsible for educational advantages observed at age 16.

## Results

### Sample characteristics

Based on questionnaire data, our sample of 1673 adolescents (54% female) were split into simultaneous multilinguals (25%), multilingual learners (19%), and monolinguals (56%). SES was indexed by a latent factor derived from parental occupation, parental education, free school meal status, and area deprivation. Cultural factors were indexed by ethnicity (white, black, Asian, mixed). Educational achievement was assessed at two time points, Standardised Assessment Tests (SATs) sat in the summer of the children’s final year of English primary school (Year 6; age 11) and GCSE examinations in English language, maths and science at the end of their final year of compulsory English secondary education (Year 11; age 16), from which two latent factors were derived. Cognition was assessed twice, once at 12 years of age and again at 14 years of age, using tests of cognitive flexibility, verbal working memory, spatial working memory, and non-verbal reasoning. A latent cognitive factor was derived from these tests to give a psychometrically robust measure^9^.

Table 1 shows a correlation matrix of these variables. It confirms the possibility of confounds in studies of multilingualism: both SES and ethnicity were correlated with multilingualism (as well as with each other), and both SES and ethnicity showed associations with educational attainment and with the cognitive factor. These associations are further unpacked using ANOVA in Supplementary Materials. These supplementary analyses showed significant interactions between ethnicity and language status on SES and age 14 cognition, highlighting the complex relationships between these variables.

**Table 1:** Correlation matrix of multilingual status, SES, ethnicity, cognitive skills, and attainment

	SES factor	White	Black	Asian	Mixed
White	**0.14**				
Black	0.01				
Asian	**–0.21**				
Mixed	0.05				
Simultaneous multilinguals	**–0.20**	**–0.26**	–0.04	**0.41**	**–0.09**
Multilingual learners	**–0.16**	**–0.10**	–0.02	**0.13**	0
T1 Cognitive flexibility	0.01	**0.08**	**–0.06**	–0.01	–0.04
T1 Verbal working memory	**0.05**	**–0.06**	–0.04	**0.09**	0.02
T1 Spatial working memory	**0.09**	0.02	**–0.1**	**0.07**	-0.01
T1 Non-verbal reasoning	**0.13**	0	**–0.11**	**0.09**	0.01
T1 Cognitive factor	**0.12**	–0.01	**–0.11**	**0.11**	0.01
T2 Cognitive flexibility	0.01	**0.08**	–0.05	0	**–0.06**
T2 Verbal working memory	**0.10**	**–0.08**	**–0.07**	**0.14**	0.03
T2 Spatial working memory	0.05	**0.05**	**–0.10**	0.03	0
T2 Non-verbal reasoning	**0.17**	0.01	**–0.15**	**0.09**	**0.06**
T2 Cognitive factor	**0.15**	–0.01	**–0.14**	**0.11**	0.04
Age 11 attainment factor	**0.23**	0.05	**–0.14**	0.05	0.04
Age 16 attainment factor	**0.26**	-0.05	**–0.15**	**0.20**	-0.01

Emboldened correlations are significant at *p* < 0.05.

Values are from parametric pairwise complete correlations. *n* varies for different pairings of variables. For each ethnicity and first language category, the reference group is all other categories of ethnicity/first language. Scores from all cognitive tasks were coded so that a higher score = better performance for ease of interpretation.

Studies that investigate multilingualism, then, are at risk of confounding the effects of SES and cultural factors unless these are explicitly controlled for. In our sample, monolinguals typically came from families more socioeconomically advantaged than the average for the sample whilst both multilingual groups came from less advantaged families than average for this sample; most monolinguals were white whilst most simultaneous multilinguals were Asian, and multilingual learners were more evenly distributed across ethnicity groups (see “Methods”).

### Educational attainment models

Filippi et al. previously reported educational differences associated with multilingualism in a larger sample drawn from this cohort^3^; here we used a reduced sample for whom longitudinal cognitive data were also available. We first confirmed that similar educational effects held for this reduced sample. We used structural equation modelling to understand associations between multilingual status and educational attainment, first at 11 years of age, then at 16 years of age, and finally considered the change from 11 to 16 years of age. In a first step, we used models excluding SES and ethnicity which would be at increased risk of confounds, to test how such confounds might create or obscure apparent effects of multilingualism. In a second step, we then ran adjusted models that controlled for SES and ethnicity. Asians were overrepresented in the multilingual sample, and this was the only ethnicity to predict higher scores on multiple cognitive measures (relative to all other ethnicities) and to positively predict attainment. We therefore employed Asian vs not Asian as our ethnicity measure to index cultural factors. All analyses were run in Rstudio using lavaan’s sem() function with full information maximum likelihood estimation (given the presence of missing data) and robust standard errors (to account for clustering in the data).

**Fig. 1:**
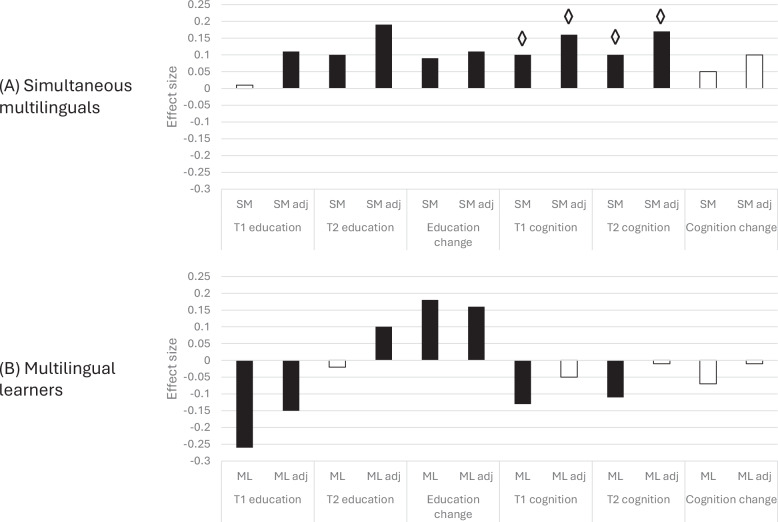
Multilingual associations with educational attainment and cognitive outcomes. Bars show standardized regression coefficients (β) from structural equation models linking language group to educational attainment and cognitive abilities. Each set of bars represents a different model specification. Panel **A** compares simultaneous multilinguals (exposed to more than one language since birth) with monolinguals; panel **B** compares children learning English as a second language (i.e., English acquired after another home language) with monolinguals. Black bars indicate statistically significant associations (*p* < 0.05) and white bars indicate non-significant associations. “Adj” denotes estimates adjusted for socioeconomic status (SES) and ethnicity. An asterisk (*) indicates a significant indirect effect (mediation) of the educational association via the cognitive measure(s). For scale, an effect size of 0.2 corresponds approximately to a 7-percentile-point difference (e.g., from the 50th to the 57th percentile).

**Fig. 2:**
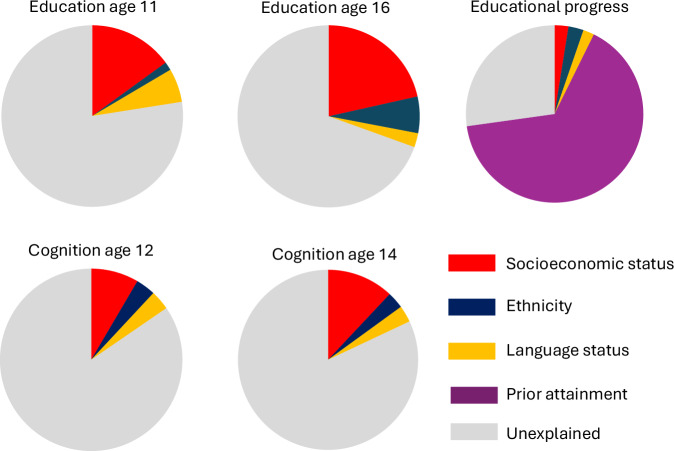
Relative contribution of SES, ethnicity, and language group to explained variance in attainment and cognition. Pie charts show the average proportion of explained variance (R²) attributable to SES, ethnicity, and language group (simultaneous multilingual vs monolingual; children learning English as a second language vs monolingual) across outcomes. For each outcome, contributions were computed as the average increase in R² when each predictor was added to the model, averaged across all possible orders of variable entry (to reduce dependence on entry sequence). The remaining portion of each pie represents unexplained variance. A pie chart is not shown for cognitive change because, in a latent change score framework, R² is zero in the baseline model without predictors; explained variance is only defined once predictors of change are included.

In the cross-sectional analysis of educational attainment at age 11, language status explained 7% of the variance (CFI = 0.98, RMSEA = 0.08). There was no significant effect of being a simultaneous multilingual (SM) versus monolingual (ϐ = 0.01, *p* = 0.816) and a small negative effect of being a multilingual learner (ML) versus monolingual (ϐ = –0.26, *p* < 0.001) on educational attainment at age 11. SM effects on age 11 attainment were, however, apparent in the adjusted model controlling for ethnicity and SES (ϐ = 0.11, *p* = 0.003). Controlling for confounds increased the size of the SM effect. ML effects were robust to the inclusion of these controls, although the effects reduced by more than a third (ϐ = –0.15, *p* < 0.001). The variance explained in age 11 attainment tripled with the addition of ethnicity and SES to the model (CFI = 0.91, RMSEA = 0.08, R^2^ = 0.22). Figure 1 illustrates these relative effect sizes. Figure 2 depicts the relative proportions of variance associated with language status, SES and ethnicity. It demonstrates that SES was the most influential predictor of educational attainment at age 11.

Only 1% of the variance in educational attainment in the cross-sectional analysis at age 16 was explained by language status (CFI = 1.00, RMSEA = 0.04). SM effects on age 16 attainment were small and positive (ϐ = 0.10, *p* < 0.001), whilst ML effects were non-significant and close to zero (ϐ = –0.02, *p* = 0.434). SM effects again increased with the inclusion of ethnicity and SES (ϐ = 0.19, *p* < 0.001), and there was evidence for a small positive effect of ML on age 16 attainment in (ϐ = 0.10, *p* < 0.001). However, SES again accounted for a greater share of the variance, and variance explained increased substantially with the addition of ethnicity and SES controls (CFI = 0.95; RMSEA = 0.08, R^2^ = 0.29).

### Cognitive outcomes models

**Fig. 3:**
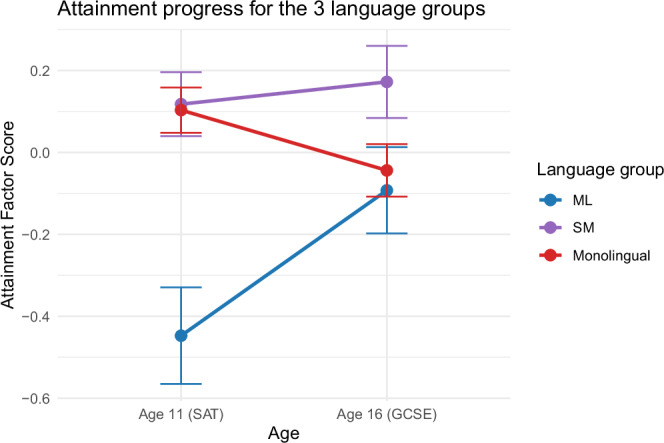
Educational attainment trajectories from age 11 to age 16 by language group. Lines show estimated educational attainment factor scores at ages 11 and 16 for monolinguals, simultaneous multilinguals, and children learning English as a second language. The figure visualises group differences at each age and the relative progress between time points. Error bars indicate 95% confidence intervals around the estimated means. Factor scores are derived from the attainment model used in the main analyses (see Methods) and are presented so that higher scores indicate higher attainment.

In the longitudinal analysis, language status and age 11 attainment now explained 68% of the variance in attainment at age 16 (with most of the variance explained by age 11 attainment; CFI = 0.92, RMSEA = 0.15). SM and ML both made more academic progress than monolinguals (SM ϐ = 0.09, *p* < 0.001; ML ϐ = 0.18, *p* < 0.001). The stronger effect for ML reflects the initial disadvantage and catchup compared to monolinguals. The results held after adjusting for SES and ethnicity (SM ϐ = 0.11, *p* < 0.001; ML ϐ = 0.20, *p* < 0.001). Adding ethnicity and SES to the model yielded a smaller increase in variance explained compared to the cross-sectional models (CFI = 0.92, RMSEA = 0.09 R^2^ = 0.73). The predictors explained a comparable amount of variance in the longitudinal model but together they accounted for a more modest proportion of the total variance than in cross-sectional models (see Fig. 2). Note that this is because the longitudinal analysis indexes effects that unfold specifically during adolescence. Multilinguals made more educational progress than monolinguals (see Fig. 3).

### Mediation analyses

Turning to cognitive ability, represented by the latent cognitive factor, we ran comparable analyses of the two cross-sectional time points, here age 12 and 14, and the change between these time points, using language status as the predictor, and additionally adjusting for the potential confounds of SES and cultural factors indexed by ethnicity. Figure 1 shows the effect sizes. SM showed a cognitive advantage at both time points, which was increased in size by controlling for SES and ethnicity, while ML showed a cognitive disadvantage at both time points which disappeared to no-difference by controlling for SES and ethnicity. Neither group showed an influence of the multilingual experience on cognitive development across this period.

For longitudinal cognitive development between 12 and 14 years, there was no significant effect of SM (ϐ = 0.06, *p* = 0.260) or ML (ϐ = –0.06, *p* = 0.282) on change in cognitive factor scores from age 12 to age 14 (CFI = 0.95, RMSEA = 0.04, R^2^ = 0.01) and this pattern held when controlling for SES and ethnicity (SM ϐ = 0.09, *p* = 0.217; ML ϐ = –0.02, *p* = 0.758). Notably, SES (ϐ = 0.29, *p* = 0.014) and ethnicity (ϐ = 0.19, *p* = 0.007) were both significant predictors of cognitive development and variance explained increased by eightfold when these factors were included in the model (CFI = 0.88, RMSEA = 0.05, R^2^ = 0.08). Cognitive development from 12 to 14 was therefore sensitive to SES and ethnicity but not to multilingual status.

### Additional analyses

Having established both an educational attainment advantage and some evidence of higher cognitive scores for SM (in the cross-sectional analyses), we can now ask whether the cognitive differences associated with the simultaneous multilingual experience may be responsible for the observed educational advantages. We used age-12 cognitive scores as a possible mediator of age-11 educational attainment, and age-14 cognitive scores as a possible mediator of educational attainment at age 16.

There was evidence for a mediation effect for educational attainment at 11. The unadjusted model was a good fit for the data (CFI = 0.95, RMSEA = 0.07, R^2^ = 0.62). Whilst the indirect effect was significant (β = 0.08 [0.03, 0.13]), confidence intervals for the total effect crossed zero because of a negative effect of SM on attainment after accounting for the indirect effect, suggesting full mediation. Controlling for ethnicity and SES, the results were also consistent with full mediation: the indirect effect was significant (β = 0.12 [0.05, 0.18]) and the direct effect was not (β = 0.00, *p*  = 0.961); CFI = 0.91, RMSEA = 0.06, R^2^ = .64. Age 14 cognitive skills were a significant partial mediator of the relationship between SM vs monolingual and age 16 attainment in both unadjusted (mediation ratio = 70%; CFI = .98, RMSEA = 0.05, R^2^ = 0.57) and adjusted models (mediation ratio = 53%; CFI = 0.94, RMSEA = 0.06, R^2^ = 0.63).

Therefore, in cross-sectional models, at least some of the multilingual educational advantage appeared to proceed via cognitive differences. However, since there was no significant relationship between SM or ML status and longitudinal change in cognitive skills from 12 to 14, such skills could not be the mediating pathway for the faster academic progress of multilinguals. Hence, the evidence for cognitive skills playing a role in multilingual educational benefits is limited. By contrast, the effect of cultural factors on educational progress was indeed partially mediated by change in cognitive skills from 12 to 14 years (see Supplementary Materials).

Our analyses concentrated on latent factors for educational attainment (across subjects), and for cognitive ability (across the tests of cognitive flexibility, verbal working memory, spatial working memory, and non-verbal reasoning) because latent factors are more psychometrically robust^9^. In supplementary analyses, we distinguished individual cognitive abilities and individual educational subjects. We found that the verbal working memory and non-verbal reasoning measures were the cognitive tests that showed the closest association with multilingual status and that educational advantages of multilingualism effects were most strongly observed in maths and science. Change in verbal working memory and non-verbal reasoning, however, were not reliably predicted by language status so once more could not serve as mediators of educational progress (Supplementary Materials).

## Discussion

The existence of educational advantages and cognitive differences in multilinguals has proved controversial both because of inconsistent evidence and the risk of confounds from factors potentially associated with multilingualism, such as differences in socioeconomic status and cultural factors. In our study, we demonstrated the presence of these confounds in our sample of 1673 UK adolescents and that they associate with key outcomes such as educational attainment and cognitive skills. Therefore, the risk of confounds is a real one in this field. However, given the relatively large sample size, we were able to distinguish the unique contribution of multilingualism controlling for these confounds, and to test for unmeasured confounds by contrasting cross-sectional and longitudinal analyses.

We observed robust educational advantages for simultaneous multilinguals compared to monolinguals at both cross-sectional time points and in longitudinal educational progress. We observed an initial educational disadvantage for multilingual learners at age 11, as they engage with an educational system in a language that they have not yet mastered, but faster progress than monolinguals that left them ahead by age 16. Controlling for the potential confounds of SES and cultural factors, here indexed by ethnicity, increased the size of the positive effects of being a simultaneous multilingual (SM)—that is, confounds may sometimes serve to mask the real effects. The robust evidence of multilingual educational advantage is of high relevance to policymakers.

The picture on cognitive skills was similar in one respect: simultaneous multilinguals showed higher cognitive scores compared to monolinguals at both ages in cross-sectional analyses, which increased in size when controlling for potential confounds of SES and ethnicity. However, in the longitudinal design, there was no difference in rate of cognitive development compared to monolinguals. While the two-year gap in cognitive measures from age 12 to 14 was shorter than the educational gap from 11 to 16, the two-year period was nevertheless enough time for both SES and ethnicity to modulate the rate of development. The lack of a multilingual effect in adolescent development implies either that for simultaneous multilinguals, the differences in cognition have already occurred and are stable by age 11, or that cognitive differences in the cross-sectional analyses actually reflect confounds that were not picked up by our measures of SES and ethnicity. For multilingual learners, lower cognitive scores were observed at both time points, but this difference disappeared when controlling for SES and ethnicity – and once more, no alteration of rate of cognitive development was observed. From a cognitive perspective, early acquisition of multiple languages may lead to higher scores, but later acquisition of a language in which the child will subsequently be educated does not appear to produce lower scores.

If the educational advantage of multilinguals were delivered by the cognitive differences, there were several opportunities for this to be revealed by mediation analyses, be it cross-sectionally in early adolescence, mid adolescence, or longitudinally in baseline-corrected models, for either simultaneous multilinguals or multilingual learners. Only cross-sectional models showed evidence of mediation via cognitive skills, and this was only the case for simultaneous multilinguals. The inference is that in the main, multilingual educational benefits are decoupled from cognitive differences (at least, of the ones we measured here) suggesting that, more broadly, the educational effects of multilingualism should be considered separately from narrow cognitive differences.

Such a conclusion would mean that the causal locus of the multilingual educational effect remains unclear. The proposal that it proceeds by a targeting specific cognitive skills such as cognitive flexibility or inhibitory control does not seem consistent with the restricted transfer effects seen in cognitive training studies^14,15^. although perhaps the duration and intensity of the multilingual experience supports farther transfer, as is sometimes argued for the effects of, for example, extensive action video game playing on attention (see 15 for discussion). The largest cognitive effect in the current study was on verbal working memory tasks, rather than cognitive flexibility. However, it may be that causal pathways are more holistic and diffuse, similar to the impact of socioeconomic status on cognition, or education on cognitive ability^16,17^. It is likely that the early educational disadvantage for multilingual learners is linked to their weaker language skills, evidenced in their age-11 English attainment (see Supplementary Materials). While we identified significant differences in English language attainment, we did not specifically test whether English acted as a mediator between multilingualism and maths or science outcomes. Future research could address this gap by exploring these indirect pathways more directly.

It is possible that advantages of multilingualism are attributable to enhanced creative capacities and better social communication skills, as suggested by studies like Kharkhurin^18^ and Gampe et al.^19,20^ (see 20 for wider discussion). Moreover, multilingualism provides access to broader cultural and social networks, potentially enriching educational experiences and outcomes. The social benefits of multilingualism might be particularly impactful during adolescence, as teenagers have heightened sensitivity to social influence^21^. It is also important to note that the Trail Making Task used to assess cognitive flexibility may not have been sufficiently sensitive to detect more subtle multilingualism-related differences. More generally, the SCAMP language measure provides categorical language status rather than finer-grained indices of proficiency and day-to-day exposure (e.g., frequency and contexts of use), which may vary substantially within groups and could moderate associations with cognition and attainment. Future longitudinal work combining linked educational outcomes with more detailed measures of language experience will be important for clarifying which aspects of bilingual experience, if any, are most closely associated with educational advantages. Finally, each country has specific distributions of SES and cultural groups, therefore the findings of this study may not apply to other countries.

In sum, multilingualism was found to be robustly associated with higher educational attainment and educational progress across adolescence in a UK cohort and may relate to higher cognitive abilities for children learning multiple languages from a young age, but the two effects are only sometimes aligned. Our findings underscore the importance of recognising and supporting multilingualism as an educational asset, with clear implications for educational policy and practice.

## Methods

### Ethics statement

This study involved secondary analyses of data from the Study of Cognition, Adolescents and Mobile Phones (SCAMP). The original SCAMP study protocol and subsequent amendments were approved by The North West Haydock Research Ethics Committee (REC reference 14/NW/0347). Ethical approval for the linkage of SCAMP data with National Pupil Database data for the purposes of RCP’s PhD project was granted by the Imperial College London Research Ethics Committee (Protocol number 14IC2067, version 4, 29/07/2014).

### Participants

The data from this study come from the regionally representative Study of Cognition, Adolescents and Mobile Phones (SCAMP), which included participants from 39 secondary schools in and around Greater London, UK, (26 state/13 independent) capturing variation in socioeconomic and demographic characteristics^22^. Two hundred and six schools with Year 7 cohort >200 (state schools) or >50 (independent schools) were initially contacted via invitation to the head teacher. The present study included 1673 pupils (54% female) who were state school educated, were present at both cognitive assessment points and had consistent multilingual information reported in the study questionnaire data and National Pupil Database (see 4 and 23 for further details of these inclusion criteria).

The sample represents greater linguistic and ethnic diversity than England and Wales as a whole. It is socioeconomically diverse, but the distribution of different indicators looks somewhat different than the national picture^23^ for comparison of a SCAMP subsample to London and England figures. These differences are somewhat to be expected because of differences between London and England and Wales as a whole.

### Design

SCAMP cognitive assessments and reporting of demographics took place when pupils were approximately 12 years old. The same cognitive measures were administered approximately 2 years later. Participants completed the tasks and questionnaires as a whole class during their IT lessons. They had the length of the scheduled class to complete the task battery. They were instructed to work through the tasks on their own, but researchers were available in case of technical issue or to clarify instructions. Cognitive tasks were always completed in the same order. The order of task presentation was determined by the perceived usefulness of the measures for answering questions about development by the SCAMP team and feedback from participants in the pilot.

Standardised national exam data from the final year of primary school (~age 11) and at the end of compulsory secondary education (~16 years) were sourced from the UK National Pupil Database.

### Multilingualism measure

As part of the SCAMP battery, participants were asked whether English was their first language, English was not their first language, or whether they learnt English at the same time as another language. These categories are henceforth referred to as monolinguals, multilingual learners (ML) and simultaneous multilinguals (SM).

### Cognitive measures

We analysed data from computerised versions of the Trail Making Task (TMT, measure = switch cost), Backward Digit Span (BDS, measure = span), Spatial Working Memory Task (SWM, measure = errors) and Cattell’s Culture Fair Test (CFT, measure = total number of items correct in Odd One Out and Complete the Patterns subtasks). These are age-appropriate tasks designed to measure cognitive flexibility (TMT), working memory (BDS and SWM) and non-verbal reasoning (CFT). These tasks, as well as the larger battery of tasks included in SCAMP, are outlined in Supplementary Materials and described in detail in refs. ^3^ and ^23^. These measures were selected for the present study as they had small amounts of missing data (due to their position in the testing battery) and were available at both assessment waves (ages ~12 and ~14), enabling longitudinal analysis. Other cognitive domains were not examined here because the most relevant measures were either not available at both waves in a comparable form and/or had substantially higher missingness in the present analytic subsample, limiting interpretability and power. Only participants who completed the cognitive tasks at UK Key Stage 3 aged 12 and Key Stage 4 aged 14 were selected.

After removing extreme outliers >3.29 SDs from the mean (99.9% of normally distributed data fall within the range of –3.29 to +3.29 SDs) and regressing out age at testing (due to a lack of correspondence between when participants completed the cognitive measures and their national exams), latent factors were derived using confirmatory factor analysis. We created an age 12 cognitive factor and an age 14 cognitive factor. Model fits were good (age 12 CFI = 1.00, RMSEA = 0.00; age 14 CFI = 1.00; RMSEA = 0.00) and all measures significantly loaded onto the latent factor (age 12 λs = .14 (TMT) to .61 (BDS); age 14 λs = .13 (TMT) to .66 (BDS); all *p* < 0.001).

### Standard assessment tests (SATs)

Pupils educated in state funded primary schools in England sit SATs in the summer of their final year of primary school (Year 6; age 11). Pupils identified as having special educational needs or social, emotional, and mental health needs can be disapplied. SCAMP pupils sat SATs in reading and maths at this time. Assessments were graded following the old system where the standard exams captured grades 3 through 5 (4 being the average and the expected standard, where a higher number reflects a higher grade). At the time the SCAMP participants took their SATs, high performing students could also be entered for level 6 papers. Since 2013, Department for Education have also collected teacher assessed grades for writing and science. These are graded on a scale of 2–6 (6 being the highest possible grade). A subset of teacher assessed marks are externally moderated.

As in ref. ^3^, reading and writing scores were combined for comparability with age 16 assessments. After regressing out age at testing (due to a lack of correspondence between when participants completed the cognitive measures and their national exams), a latent factor was derived using confirmatory factor analysis. The model was just identified but all measures significantly and strongly loaded onto the latent factor (λ_maths_ = .76; λ_English_ = .83; λ_science and English_ = .83; all p < .001).

### General certificate of secondary education (GCSE)

All English pupils sit GCSE examinations in English language, maths and science at the end of their final year of compulsory secondary education (year 11; age 16), hence our choice of these subjects as outcome measures. GCSEs are graded on a scale of 1–9 (9 being the highest grade possible, equivalent to an A* in the old grading system), where a grade of 4 (old grade C) is the pass mark. Students may sit foundation or higher papers, depending on their predicted grades; foundation papers are less academically challenging and cover the grades 1–5, whilst higher papers cover grades 3–9. Grades are calculated as a weighted average of pupils’ performance across the different papers for that subject and the grade boundaries are dependent on the distribution of performance for pupils in that year group.

After regressing out age at testing (due to a lack of correspondence between when participants completed the cognitive measures and their national exams), a latent factor was derived using confirmatory factor analysis. The model was just identified but all measures significantly loaded onto the latent factor (λ_maths_ = 0.91; λ_English_ = .75; λ_science_ = 0.97; all *p* < 0.001).

### Socioeconomic status

Socioeconomic status was treated as a factor of the available indicators for this cohort: number of university educated parents (0–2), parental highest occupational prestige (0–8, where 8 = highest; based on ONS classification system), Free School Meals status (yes/no; an indicator of household poverty), and area level deprivation (1–5; based on the Carstairs index applied to Census data). A one factor model of socioeconomic status was shown to have acceptable fit to the data and to significantly predict cognitive skills in ref. ^23^. For more detail on these indicators please see ref. ^23^.

Language groups significantly differed on average SES factor scores, F(1, 1671) = 163.40, *p* < 0.001. Monolinguals typically came from families more socioeconomically advantaged than the average for the subsample (M = 0.23, SD = 0.70), whilst both multilingual groups came from less advantaged families than average for this subsample (SM M = –0.25, SD = 0.72; ML M = –0.23, SD = 0.63).

### Ethnicity

During the initial SCAMP testing sessions, participants were asked to indicate which of the following ethnic groups they belonged to: English, Welsh, Scottish, Northern Irish, or British / Irish / Gypsy or Irish Traveller / any other White background / White and Black Caribbean / White and Black African / White and Asian / any other Mixed or Multiple ethnic background / Indian / Pakistani / Bangladeshi / Chinese / any other Asian background / African / Caribbean / any other Black, African or Caribbean background / Arab / any other ethnic group. From these options, responses were collapsed into five categories—white / black / Asian / mixed / other. In this study, we take ethnicity to be a marker for cultural factors. Therefore, the other option (<10% of responses) was not included as it as a factor as it may not represent a homogenous group of pupils.

**Fig. 4:**
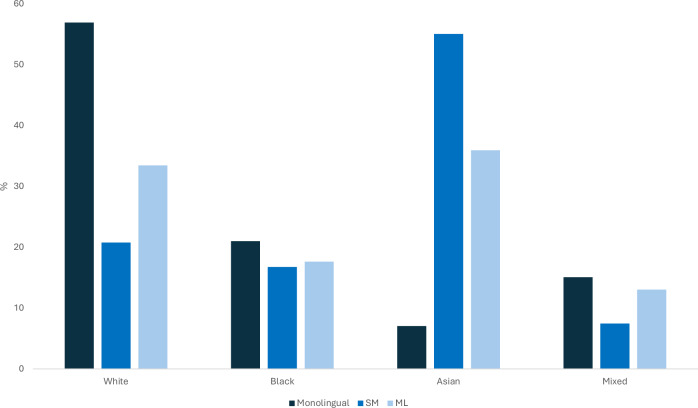
Ethnic composition of the sample by language group. Bars show the percentage of participants in each ethnicity category within each language group (monolinguals, simultaneous multilinguals, and children learning English as a second language). Percentages are calculated within language group so that each group sums to 100%. The figure illustrates differences in ethnic composition across language groups, which is relevant to the covariate adjustments reported in the main analyses.

Ethnicities were not evenly distributed across the language groups (X^2^ (6) = 378.99, *p* < 0.001). Figure 4 shows that most monolinguals were white whilst most SMs were Asian.

## Supplementary information

Supplementary materials

## Data Availability

SCAMP data are not publicly available. However, some data can be shared on request subject to approval by the SCAMP Data Access Committee. Data access requests should be directed to Dr Mireille B Toledano (Principal Investigator; m.toledano@imperial.ac.uk).

## Code availability

The code for the analyses described in this paper will be made available on OSF.
