# Supplementary material for: Multilingualism educational attainment and cognitive development in UK adolescents
Source: NPJ Sci Learn. 2026 Apr 1;11:28. doi: 10.1038/s41539-026-00411-z (PMC13212884; doi:10.1038/s41539-026-00411-z)
Supplement: Supplementary file 1 — Supplementary materials [file 41539_2026_411_MOESM1_ESM.pdf]

Supplementary materials

1. Description of tests

2. Measures of socioeconomic status

3. Ethnicity associations with first language status, cognitive performance, attainment and SES

4. English language performance across groups

5. Are SES and cultural effects on academic progress mediated by change in cognitive skills?

6. Subject-specific and cognitive-test-specific effects

5.1. Are SM effects being hidden by grouping cognitive skills together?

5.2 Attainment-measure-specific effects

1. Description of tests

In the SCAMP study (scampstudy.org), cognitive tasks and questionnaires were designed and completed through Psytools software (Delosis Ltd; https://www.delosis.com/psytools/) as a whole class during the participants' IT lessons in their school computer suites. Tasks which are generally administered as pen and paper measures were adapted to be run on computers (see Toledano et al., 2018 for details of the complete task and questionnaire battery). Cognitive data for 11–12-year-olds were collected in 2014-16, while those for 13–14-year-olds were collected in 2016-18. The cognitive tests used were as follows:

Trail-Making Test (TMT). The TMT (Tombaugh, 2004) measured *cognitive flexibility*. In the letters condition, dots were labelled with letters and participants had to click them in alphabetical order. In the switching condition, dots were labelled with a number or a letter and participants were required to click these in alternating alphabetical and numerical sequences. The measure of task performance is the residual of the time to complete the numbers/letters condition after covarying the time to complete the letters condition. This measure reflects relative response time on the switching block compared to the single sequence block, a higher value reflects a greater switch cost. See Figure S1 for example stimuli.

Backward Digit Span (BDS). *Verbal working memory* was measured with a variant of the BDS task (Dumontheil & Klingberg, 2012). Single digit numbers were presented visually individually, participants were required to reproduce the number sequence in reverse order using a numerical response pad displayed on screen. Sequence length could vary between 2 and 9 and was increased or decreased incrementally following the staircase/Levitt procedure (Levitt, 1971). The measure on this task was the average of the mean sequence length of trials with a correct response and the mean sequence length of trials with an incorrect response. See Figure S1 for example stimuli.

Spatial Working Memory Task (SWM). The SWM was used to measure *spatial working memory and planning* and was adapted from the Cambridge Neuropsychological Testing Automated Battery (Luciana & Nelson, 2002) task of the same name. The task required participants to click on ringing telephones to answer them. Participants had to work out which phone was ringing at a given time and answer that phone, while recalling

which ones had already rung to avoid selecting the same phone twice. Four levels played out in ascending order of difficulty (4, 6, 8, then 10 phones). Measures were the total number of errors and a strategy score, which reflects planning and is the number of excess switches of the starting location on each search at levels 6, 8 and 10 (this is based on the assumption that starting the search in the same location on each trial, except if this phone has already rung, would aid performance and reflect planning). A higher strategy score reflects a less efficient strategy. We used the measure with the best psychometric properties which was total number of errors. See Figure S1 for example stimuli. In correlation matrices, this variable is reverse coded for clarity of presentation.

Cattel's Culture Fair Test (CFT). We used stimuli from the Cattel's Culture Fair Test (Cattell et al., 1960) Form A, Scale 2 as a measure of *non-verbal reasoning*, specifically the Odd One Out and Complete the Pattern subtests. Participants had three minutes for each task to complete as many trials as possible (up to 14 for Odd One Out; up to 11 for Complete the Pattern). The measure on this task was the total number of correct trials on the two subtests. See Figure S2 for example stimuli.

Figure S1

Example cognitive task stimuli. (a) Trail Making Task letters (top) and switching conditions (bottom). (b) Example of a Backwards Digit Span task trial. (c) Example of a screen capture from the Spatial Working Memory task showing feedback given for a correct selection.

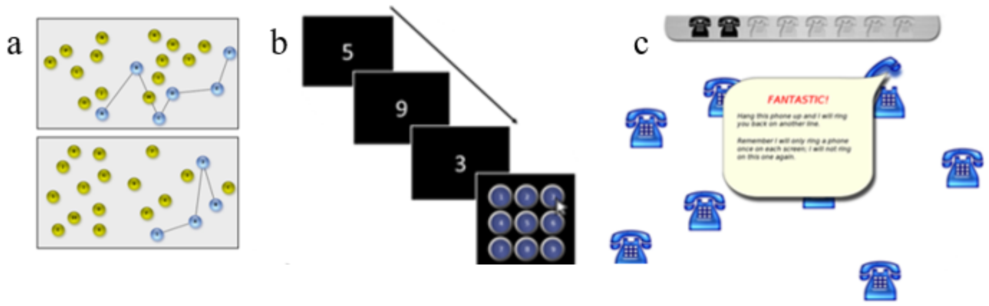

Figure S2

Non-verbal reasoning task stimuli. (a) Example Odd One Out task trial. (b) Example Complete the Pattern task trial.

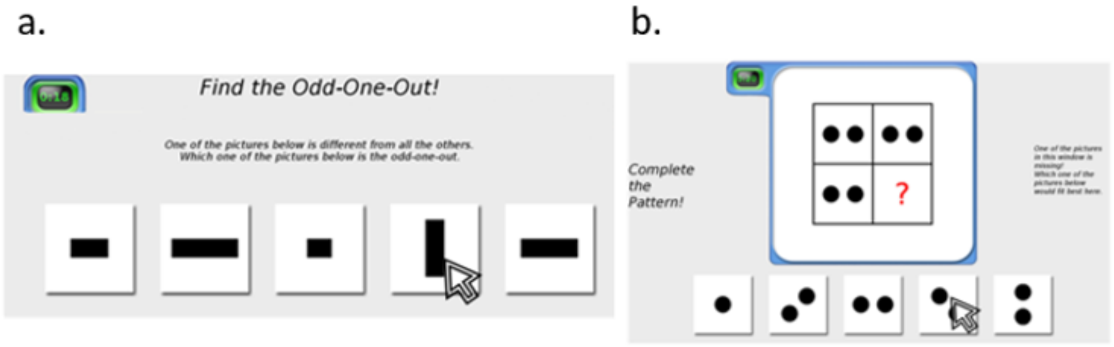

2. Measures of socioeconomic status

The following indices were used to assess socioeconomic status:

Number of parents who attended university. Participants were asked "*Did your mother attend university?*" and "*Did your father attend university?*" These variables were found to have very high rates of missingness at T1 (34-44%). This rate dropped by 6% for each parent at T2 and that there was very high consistency between the answers given at both time points (84% for mothers and 85% for fathers). We assumed the T2 reports to be more accurate, as pupils were older, and so used T2 values instead of T1 values, except in cases where there was a response given at T1 but not T2, when the T1 response was kept.

In the SCAMP subsample, it was much more common to have 0 or 2 university educated parents as opposed to 1, likely due to assortative mating (e.g., see Robinson et al., 2017). We therefore created a total number of parents who attended university variable (0, 1 or 2) to avoid multicollinearity issues and reduce missing data from single parent families. Research has shown that parental highest level of education predicts education promoting beliefs and behaviours in the home environment, irrespective of which parent is more highly educated (Davis-Kean, 2005). However, we did not adopt the popular dominance approach because this has been shown to underestimate the effect of parental education on child educational attainment when parents are educated to similar levels (Ballarino et al., 2021).

Parental highest occupation. We took the highest occupation classification of the pupil's parents as the index, assuming that, like parental education, which parent has the highest occupational prestige has minimal relevance to child outcomes. The classification is shown in Table S1.

Free School Meals (FSM) status. Pupils with English nationality aged 5-16 are eligible to receive FSM if they come from very low-income families. Since 2010, the threshold below which families are eligible to claim has been capped at £16,190 per household per annum (pre-tax) for families in receipt of Child Tax Credits (less than one adult full-time minimum wage in England; GOV.UK, 2024). There are also some special categories of pupils that come from families with no recourse for public funding who are eligible.

Area level deprivation. Area level deprivation was scored on a scale of 1-5, based on the Carstairs index applied to Census data (Carstairs & Morris, 1990).

Table S1 shows a comparison of the SCAMP sub-sample used in the current study to national figures on SES indices and ethnicity. The sub-sample had a smaller proportion of participants who were white or had English as their first language, a skew towards higher occupations (note we took the highest occupation of the two parents in our sample), higher area level deprivation, and fewer free school meal recipients. This reflects, in part, differences between the Greater London area and England as a whole.

Table S1

Demographics of the Included Sample

	SCAMP subsample *n* (%)	*England figures*
**First language**	**1,673**	
English	936 (56%)	>90%[a]
English learnt at the same time as another language	412 (25%)	

Not English	325 (19%)	N/A
**Ethnicity**	**1,556**	
White (British, Irish, other)	683 (44%)	82%[b]
Black (Caribbean, African, other)	301 (19%)	4%
Asian (Indian, Pakistani, Bangladeshi, Chinese, other)	372 (24%)	9%
Any mixed race	200 (13%)	3%
**Number of university-educated parents**	**1,203**	
0	451 (37%)	Not
1	265 (22%)	available[c]
2	487 (31%)	
**Parent highest occupation**	**1,452**	
0 - Never worked and long-term unemployed	0 (0%)	9%[d]
1 - Routine occupations	57 (4%)	12%
2 - Semi-routine occupations	143 (10%)	11%
3 - Lower supervisory & technical occupations	83 (6%)	5%
4 - Small employers & own account workers	268 (18%)	11%
5 - Intermediate occupations	137 (9%)	11%
6 - Lower managerial, administrative & professional occupations	287 (20%)	20%
7 - Higher professional occupations	368 (25%)	
8 - Large employers, higher managerial & administrative occupations	109 (8%)	13%
**Area-level deprivation**	**1,626**	
1 – least deprived	111 (7%)	20%[e]
2	244 (15%)	20%

3	307 (19%)	20%
4	395 (24%)	20%
5 – most deprived	569 (35%)	20%
**Free school meals status**	**1,673**	
Eligible	277 (17%)	24%[f]
Not eligible	1396 (83%)	76%

Notes. Percentages are expressed as the proportion of included participants with data for that measure that belong to a given category. Ethnicity classifications are based on the UK Office for National Statistics classification system: https://www.ons.gov.uk/methodology/classificationsandstandards/measuringequality/ethnicgroupnationalidentityandreligion

[a] 2011 Census results for England and Wales (GOV.UK, 2018).

[b] 2021 Census results for England and Wales (GOV.UK, 2022).

[c] According to 2021 Census estimates, 34% of the English population over 16 are university educated (Office for National Statistics, 2023).

[d] 2021 Census data on all adults aged over 16 (Office for National Statistics, 2022).

[e] The expected distribution for area-level deprivation is 20% in each category as the index divides the region of interest into quintiles.

[f] State school pupils in the 2022/23 academic year (GOV.UK, 2023).

3. Ethnicity associations with first language status, cognitive performance, attainment and SES

Table S2

Estimated marginal means, and significance values from ANOVAs looking at how ethnicity associates with cognitive skills, attainment, language status and SES.

	Ethnicity					Language group				Interaction
	p-value for main effect	White	Black	Asian	Mixed	p-value for main effect	SM	ML	Mono-lingual	p-value for interaction
**SES factor**	***	-0.07 (0.4)	-0.07 (0.05)	-0.17 (0.04)	0.04 (0.06)	***	-0.17 (0.05)	-0.21 (0.04)	0.18 (0.03)	*
**T1 Cognitive factor**	***	-0.01 (0.04)	-0.19 (0.05)	0.15 (0.04)	0.02 (0.06)	***	0.12 (0.05)	-0.18 (0.05)	0.04 (0.03)	n.s.

**T2 Cognitive factor**	***	-0.03 (0.04)	-0.27 (0.05)	0.16 (0.04)	0.13 (0.07)	***	0.11 (0.05)	-0.15 (0.05)	0.04 (0.03)	*
**Age 11 attainment factor**	***	-0.03 (0.05)	-0.35 (0.06)	0.10 (0.05)	0.03 (0.08)	***	0.07 (0.06)	-0.40 (0.06)	0.14 (0.04)	n.s.
**Age 16 attainment factor**	***	-0.06 (0.05)	-0.29 (0.06)	0.41 (0.06)	-0.01 (0.08)	n.s.	0.08 (0.06)	-0.10 (0.06)	0.06 (0.04)	n.s.

Significant interactions between ethnicity and language status highlighted in this table were unpacked by comparing estimated marginal means across groups.

Interaction effect on SES:

All ethnicities except mixed were reliably negatively associated with SES in MLs (EM mean white = -.27, SE = .07; EM mean black = -.23, SE = .10; EM mean Asian = -.31, SE = .07). White (EM mean = -.17, SE = .08) and Asian (EM mean = -.40, SE = .05) but not black and mixed

were reliably negatively associated with SES in SMs. However, all ethnicities were positively associated with SES in monolinguals (EM mean white = .23, SE = .03; EM mean black = .13, SE = .05; EM mean Asian = .20, SE = .09; EM mean mixed = .15, SE = .06).

Interaction effect on age 14 cognition:

Mixed ethnicity was never reliably associated with cognition. There was a reliable positive effect of Asian ethnicity in SM (EM mean = .17, SE = .05) and monolingual groups (EM mean = .21, SE = .09) but not MLs. Black ethnicity was negatively associated with cognition in monolinguals (EM mean = -.15, SE = .05) and MLs (EM mean = -.52, SE = .11) but not SMs. White ethnicity was negatively associated with cognition in MLs (EM mean = -.31, SE = .08), positively in SMs (EM mean = .18, SE = .08) and not reliably associated in monolinguals.

4. English language performance across groups

One possible explanation of educational differences among language groups is their skill in English. Particularly for multilingual learners, this may provide a barrier to accessing educational content. The SCAMP cohort does not provide direct measures of vocabulary, but English skills can be gauged by educational attainment in English at KS2 and KS4. Figure S3 shows these data, confirming the lower performance of ML at KS2 (age 11) but smaller differences at KS4 (age 16).

Figure S3. Performance in English per language group in age-11 and age-16 educational tests. SM = simultaneous multilingual; ML = multilingual learner.

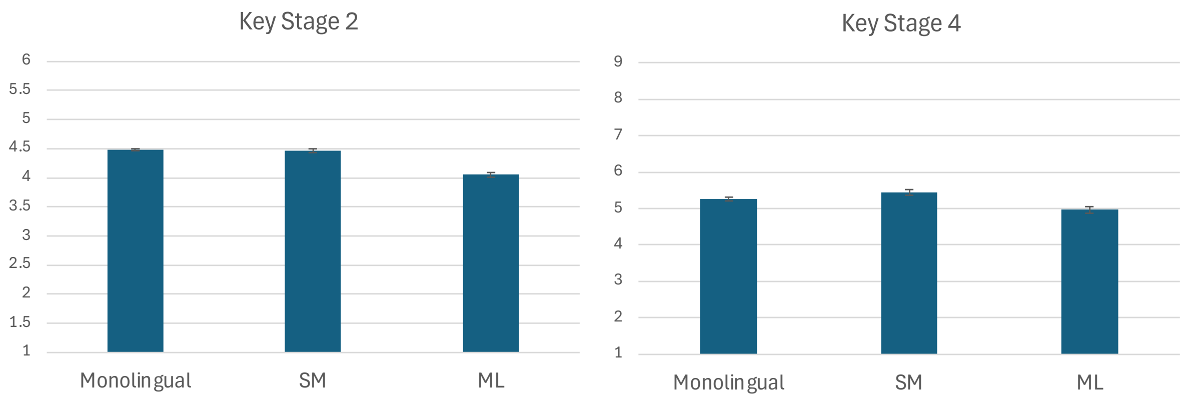

Note. Error bars give SEs.

5. Are SES and cultural effects on academic progress mediated by change in cognitive skills?

Analyses (described in the main text) showed that both SES and cultural factors were significant predictors of change in cognitive skills from 12 to 14 years of age. We therefore extended this model to test whether these characteristics might associate with academic progress via change in cognitive skills. Language status, cultural factors

and SES were simultaneously entered as predictors of change in cognitive skills and age 16 attainment (controlling for age 11 attainment). There was no significant direct or indirect effect of SES on attainment progress (indirect effect β = .03 [-.02, .08]; direct effect β = .17 [-.05, .21]). By contrast, a significant portion (17%) of the association between cultural factors and attainment progress was mediated by change in cognitive skills (indirect effect β = .03 [.01, .07]; direct effect β = .15 [.03, .35]).

6. Subject-specific and cognitive-test-specific effects

We examined the correlations between individual cognitive tests and educational attainment in the different subjects of English, Maths, and Science. Associations between cognitive measures at age 12 and age 11 educational attainment are shown in Table S3. Associations between cognitive measures at age 14 and age 16 educational attainment are shown in Table S4.

Table S3. Correlation matrix of the associations between different cognitive measures at age 12, multilingual status, and age 11 educational attainment in different subjects. Emboldened correlations are significant at p < .05. A higher score = better performance on all measures.

	ML vs monolinguals	SM vs monolingual	English	Maths	Science
ML vs monolingual	-	-	**-0.28**	**-0.12**	**-0.22**
SM vs monolingual	-	-	0.02	**0.07**	0.00
TMT	**0.07**	**-0.07**	-0.01	**0.07**	0.00
BDS	**-0.07**	**0.1**	**0.36**	**0.38**	**0.31**
SWM	**-0.13**	0.01	**0.25**	**0.31**	**0.24**
CFT	**-0.05**	**0.06**	**0.36**	**0.45**	**0.38**

N.B. Values are from parametric pairwise complete correlations. n varies for different pairings of variables.

Table S4. Correlation matrix of the associations between different cognitive measures at age 14, multilingual status, and age 16 educational attainment in different subjects. Emboldened correlations are significant at p < .05. A higher score = better performance on all measures.

	ML vs monolinguals	SM vs monolingual	English	Maths	Science
ML vs monolingual	-	-	**-0.07**	-0.02	-0.01
SM vs monolingual	-	-	0.05	**0.11**	**0.10**
TMT	**0.07**	-0.04	**0.06**	**0.10**	**0.07**
BDS	**-0.06**	**0.11**	**0.36**	**0.45**	**0.42**
SWM	**-0.12**	0.03	**0.25**	**0.33**	**0.30**
CFT	**-0.07**	0.04	**0.34**	**0.47**	**0.43**

N.B. Values are from parametric pairwise complete correlations. n varies for different pairings of variables.

CFT was associated with attainment across subjects and timepoints but only associated with SM at T1. Backwards digit span, the measure of verbal working memory, significantly associated with multilingual status at both time points. It also predicted attainment across the core subjects at both timepoints. The only other cognitive measure associated with both SM and attainment at a given timepoint was TMT at age 12; the negative association between this measure of cognitive flexibility and SM (**Table S3**) suggests it also does not underpin SM academic advantages.

6.1. Are SM effects being hidden by grouping cognitive skills together?

A mediation model testing whether SM predicts attainment at age 11 via age 12 CFT showed evidence of mediation (unadjusted β = .03 [.003, .05], full mediation; adjusted β = .05 [.02, .08], 45% mediated). However, mediation analysis looking at change in CFT as a predictor of academic progress were not indicated; SM was not a significant predictor of change in CFT (unadjusted β = -.02, p = .350; adjusted β = .04, p = .198) when regressing SM, ML, and performance on other cognitive measures (and SES and Asian in adjusted models) onto age 14 CFT, controlling for age 12 CFT. The data were therefore not consistent with improvements in non-verbal reasoning driving the educational benefits of multilingualism.

Age 12 BDS was a significant mediator of the association between SM and age 11 attainment (indirect effect: unadjusted β = .04 [.02, .06]; adjusted β = .04 [.02, .07]). Unadjusted models indicated full mediation and adjusted models indicated that 36% of the SM effect was mediated through BDS. Age 14 BDS was a significant mediator of the association between SM and age 16 attainment (indirect effect: unadjusted β = .04 [.02, .07]; adjusted β = .04 [.02, .07]). Unadjusted and adjusted models indicated partial mediation (mediation ratios = 40% and 21%, respectively).

SM was a significant predictor of change in BDS when regressing SM, ML onto age 14 BDS, controlling for age 12 BDS and performance on other cognitive measures (unadjusted β = .05, p = .030). However, this effect was marginal in the adjusted model (β = .06, p = .048); the model yielded a p-value that would not survive even the least conservative corrections for multiple comparisons. The data therefore provide little

evidence for improvements in verbal working memory driving the educational benefits of multilingualism.

6.2 Attainment-measure-specific effects

SM reliably predicted attainment in maths at age 11, maths and science at 16 and academic progress in all subjects (see **Table S5**). All effects were small but generally larger for maths and science than for English. Since neither change in the cognitive factor, CFT or BDS were reliably predicted by SM, there is no suggestion that the SM academic advantage in any subject is related to effects of SM on cognitive development. n.s. non-significant; * p < .05; ** p < .01; *** p < .001

Table S5. Relationship between SM and attainment in maths and science at age 11, age 16, and progress from 11 to 16. Data show standardised beta coefficients.

	English	Maths	Science
	Unadjusted/adjusted β		
Age 11	-.02 (n.s.) / .07*	.06* / .12***	-.01 (n.s.) / .09**
Age 16	.05 (n.s.) / .15***	.10*** / .18***	.09*** / .18***
Progress	.04* / .10***	.07*** / .10***	.07*** / .12**

Supplementary Material References

Ballarino, G., Meraviglia, C., & Panichella, N. (2021). Both parents matter. Family-based educational inequality in Italy over the second half of the 20th century. *Research in Social Stratification and Mobility*, *73*, 100597. https://doi.org/10.1016/j.rssm.2021.100597

Carstairs, V., & Morris, R. (1990). Deprivation and health in Scotland. *Health Bulletin*, *48*(4), 162–175.

Cattell, R. B., Cattell, A. K. S., & Institute for Personality and Ability Testing. (1960). *Measuring intelligence with the Culture Fair Tests*. Institute for Personality and Ability Testing.

Davis-Kean, P. E. (2005). The influence of parent education and family income on child achievement: The indirect role of parental expectations and the home environment. *Journal of Family Psychology*, *19(2)*, 294–304. https://doi.org/10.1037/0893-3200.19.2.294

Dumontheil, I., & Klingberg, T. (2012). Brain activity during a visuospatial working memory task predicts arithmetical performance 2 years later. *Cerebral Cortex*, *22(5)*, 1078–1085. https://doi.org/10.1093/cercor/bhr175

GOV.UK. (2018). *Socioeconomic status*. https://www.ethnicity-facts-figures.service.gov.uk/uk-population-by-ethnicity/demographics/socioeconomic-status/latest

GOV.UK. (2022). *Population of England and Wales*. https://www.ethnicity-facts-figures.service.gov.uk/uk-population-by-ethnicity/national-and-regional-populations/population-of-england-and-wales/latest

GOV.UK. (2023). *Schools, pupils and their characteristics, Academic year 2022/23*. https://explore-education-statistics.service.gov.uk/find-statistics/school-pupils-and-their-characteristics/2022-23#dataBlock-c3f854ee-256e-4842-b89d-e1cb53110af5-tables

Levitt, H. (1971). Transformed up-down methods in psychoacoustics. Journal of the *Acoustical Society of America*, *49*, 467–477. https://doi.org/10.1121/1.1912375

Luciana, M., & Nelson, C. A. (2002). Assessment of neuropsychological function through use of the Cambridge Neuropsychological Testing Automated Battery: Performance in 4- to 12-year-old children. *Developmental Neuropsychology*, *22(3)*, 595–624. https://doi.org/10.1207/S15326942DN2203_3

Office for National Statistics. (2022). *Industry and occupation, England and Wales*. https://www.ons.gov.uk/employmentandlabourmarket/peopleinwork/employmentandemployeetypes/bulletins/industryandoccupationenglandandwales/census2021

Office for National Statistics. (2023). *Education, England and Wales*. https://www.ons.gov.uk/peoplepopulationandcommunity/educationandchildcare/bulletins/educationenglandandwales/census2021

Robinson, M. R., Kleinman, A., Graff, M., Vinkhuyzen, A. A., Couper, D., Miller, M. B., ... & Visscher, P. M. (2017). Genetic evidence of assortative mating in humans. *Nature Human Behaviour, 1*(1), 0016. http://dx.doi.org/10.1038/s41562-016-0016

Toledano, M., B., Mutz, J., Röösli, M., Thomas, M., S, C., Dumontheil, I., & Elliott, P. (2018). Cohort Profile: The Study of Cognition, Adolescents and Mobile Phones (SCAMP). *International Journal of Epidemiology*, *48*(1), 25–26l. https://doi.org/10.1093/ije/dyy192

Tombaugh, T. N. (2004). Trail Making Test A and B: Normative data stratified by age and education. *Archives of Clinical Neuropsychology*, *19(2)*, 203–214. https://doi.org/10.1016/S0887-6177(03)00039-8
